# Successful surgery for secondary aortoduodenal fistula based on ^18^F-fluorodeoxyglucose positron emission tomography/computed tomography findings

**DOI:** 10.1016/j.jvscit.2023.101162

**Published:** 2023-03-21

**Authors:** Satoshi Sakakibara, Takayuki Shijo, Koichi Maeda, Kizuku Yamashita, Toru Ide, Ryota Matsumoto, Kazuo Shimamura, Shigeru Miyagawa

**Affiliations:** Department of Cardiovascular Surgery, Osaka University Graduate School of Medicine, Osaka, Japan

**Keywords:** Aortoduodenal fistula, ^18^F-fluorodeoxyglucose PET, Infection mapping

## Abstract

Secondary aortoduodenal fistula (sADF) is a critical late complication of abdominal aortic repair, requiring complete excision of the infected prosthesis. However, this is a highly invasive procedure for the elderly. We describe a case of sADF repair in a 76-year-old woman. Through ^18^F (fluorine-18)-fluorodeoxyglucose (FDG) positron emission tomography/computed tomography mapping, focal high FDG uptake at the sADF site, right medial limb, and ligated left lateral limb of the prosthesis was detected. The duodenal and prosthetic grafts were partially resected. The proximal and distal anastomotic segments, with no FDG uptake, were retained. The abdominal aorta was reconstructed using a bovine pericardium roll and femorofemoral bypass. Thus, FDG positron emission tomography/computed tomography mapping of the infection site could help in such cases.

Secondary aortoduodenal fistula (sADF) is a serious late complication of graft replacement of the abdominal aorta, associated with mortality of 26%.^1^ Previous reports have suggested duodenectomy and complete excision of the infected prosthesis.^2^ However, total extensive debridement in elderly and other high-risk patients is an extremely invasive procedure.^3^ Few investigators have described a technique whereby the prosthetic graft can be preserved, with feasible outcomes.^4^^,^^5^ Therefore, mapping the focus of infection is needed to determine the minimum adequate surgery for high-risk patients. The use of ^18^F (fluorine-18)-fluorodeoxyglucose (FDG) positron emission tomography/computed tomography (PET/CT) can enable more precise and less radical surgical repair in such cases. In the present case report, we describe a case of sADF repair using FDG-PET/CT. The patient provided written informed consent for the report of her case details and imaging studies.

## Case report

**Fig 1:**
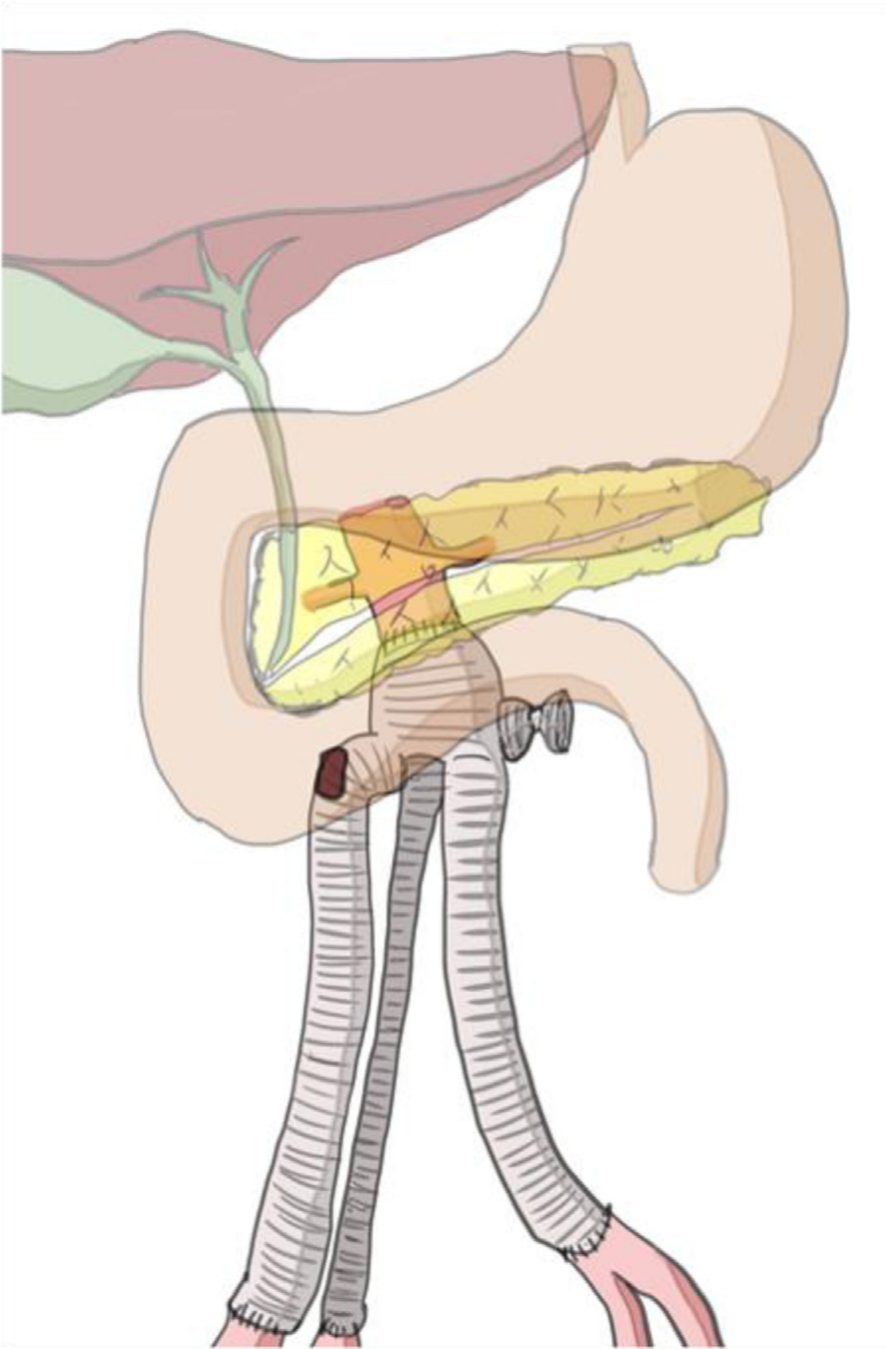
In the last surgery, the proximal anastomosis was located just below the renal arteries. The right external and internal iliac arteries were reconstructed, and the right common iliac artery was resected. The left lateral limb of the prosthesis graft was ligated.

Eight years before her referral to our hospital, a 76-year-old woman underwent endovascular abdominal aortic aneurysm repair followed, 1 year later, by infected graft replacement with a quadfurcated prosthesis (Gelsoft double bifurcate; Vascutek, Renfrewshire, Scotland, UK). In the last surgery, the proximal anastomosis was located just below the renal arteries, the right common iliac artery was resected, and the external and internal iliac arteries were reconstructed. Anastomosis was performed at the distal left common iliac artery with the left medial limb of the quadfurcated graft, and the left lateral limb was ligated (Fig 1).

**Fig 2:**
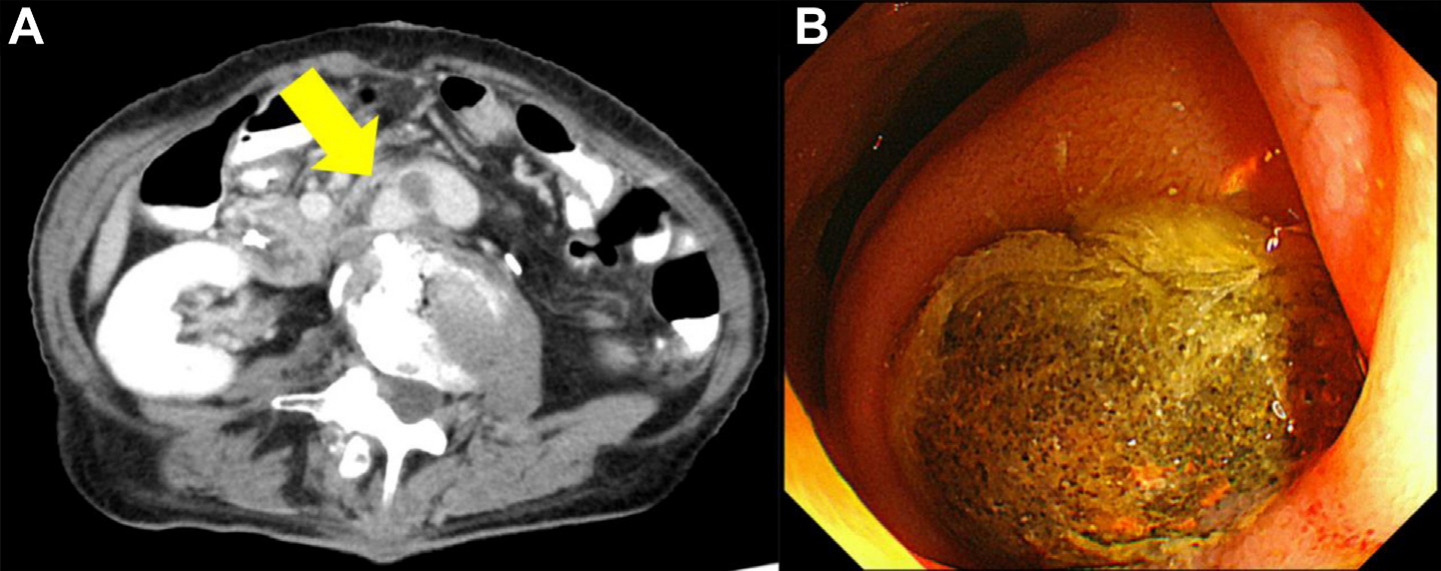
**A,** Preoperative computed tomography (CT) scan showing compression of the duodenum by the graft, with air bubbles around the graft (*yellow arrow*). **B,** Gastrointestinal fiberoptic endoscopy showing the exposed graft in the duodenal lumen.

**Fig 3:**
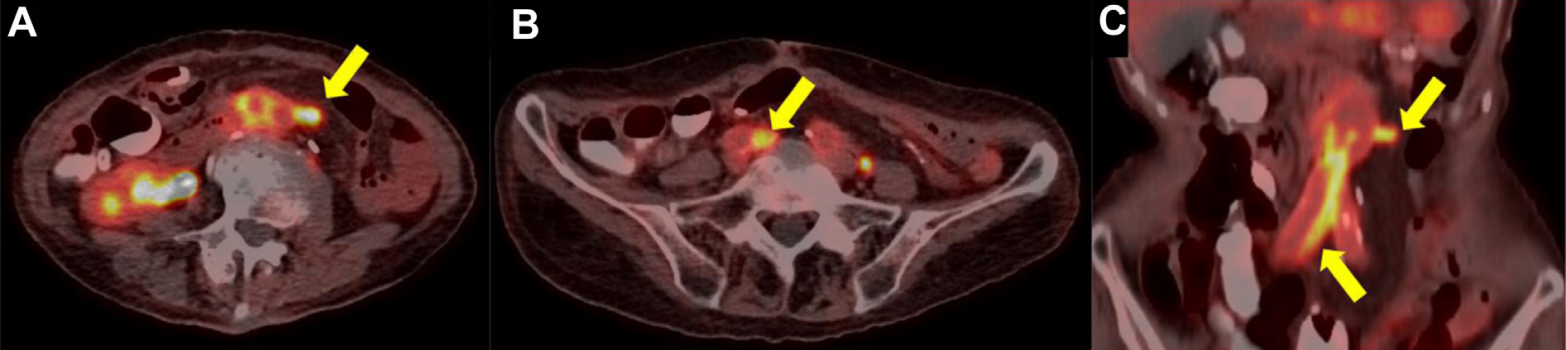
**A-C,** Preoperative ^18^F (fluorine-18)-fluorodeoxyglucose (FDG) positron emission tomography/computed tomography (PET/CT) scans showing abnormal ^18^F-FDG uptake (*yellow arrows*).

Before referral, the patient had developed exertional dyspnea and loss of appetite. Blood tests showed a normal white blood cell count (3250 cells/μL), moderately elevated serum C-reactive protein (2.03 mg/dL), and negative blood culture. However, CT revealed duodenal compression at the right lateral limb with surrounding air bubbles and right medial limb occlusion (Fig 2, *A*). Gastrointestinal fiberoptic endoscopy revealed a duodenal mucosal defect located ∼3 cm distal to the ampulla of Vater (Fig 2, *B*). Therefore, the patient was diagnosed with an sADF between the third part of the duodenum and right lateral limb. FDG-PET/CT revealed focal high FDG uptake at the following three sites: the sADF site (maximum standard uptake value [SUV_max_], 6.32), right medial limb (SUV_max_, 8.10), and ligated left lateral limb of the prosthesis (SUV_max_, 10.20; Fig 3). In contrast, no FDG uptake was found at the main trunk (SUV_max_, 2.69), right lateral limb (SUV_max_, 2.55), or left medial limb (SUV_max_, 2.43). For total prosthesis excision, extensive dissection and suprarenal aortic clamping would have been required. Thus, based on the FDG uptake, we reduced the invasiveness by preserving the proximal and left distal prosthesis segments. Additionally, to mitigate late recurrent infection related to the previously harvested omentum, we used a bovine pericardial roll for antimicrobial material.

**Fig 4:**
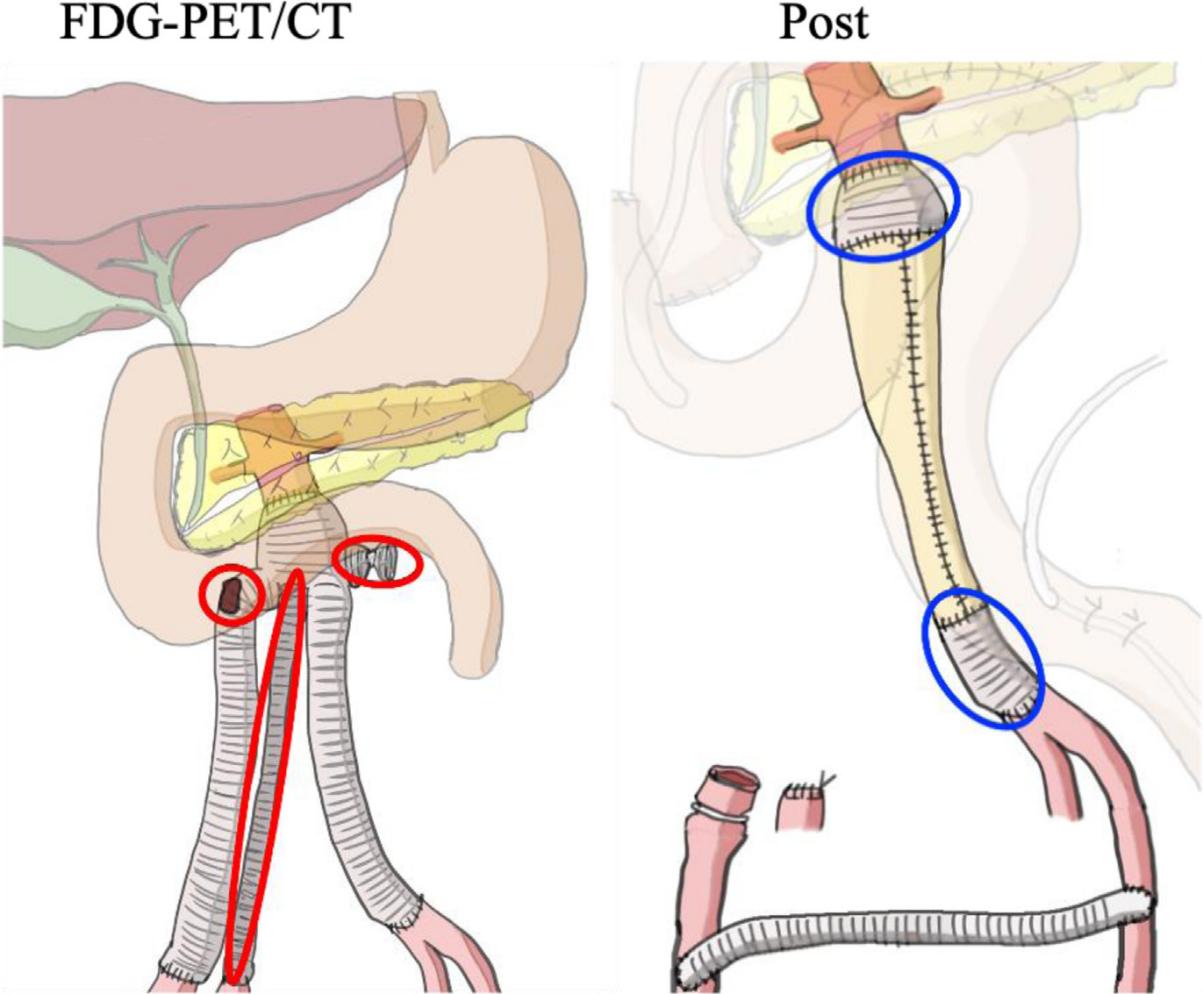
Images showing abnormal ^18^F (fluorine-18)-fluorodeoxyglucose (FDG) uptake (*red circles*) and preserved graft (*blue circles*).

First, we performed a femorofemoral (FF) bypass with a ringed, 8-mm, expanded polytetrafluoroethylene graft (PROPATEN; W.L. Gore & Associates, Flagstaff, AZ), followed by a midline laparotomy. The intra-abdominal adhesions were severe. The duodenum was mobilized medially using the Kocher maneuver and laterally from the Treitz ligament without injury. Next, the proximal third duodenal portion and proximal jejunum were stapled and cut. After prosthesis exposure, the fistula was resected en bloc, including the right medial and lateral limbs and duodenum. Blood flow in the right lower extremity was maintained through the FF bypass. Subsequently, the graft was replaced from the main trunk to the left medial limb using bovine pericardium (W.L. Gore & Associates). Finally, the jejunum was lifted toward the remaining duodenum and stomach, and a Braun anastomosis was performed (Fig 4). The operation and lower extremity ischemic times were 702 minutes and 29 minutes, respectively. The blood loss was 1250 mL.

Methicillin-resistant *Staphylococcus epidermidis* and *Candida albicans* were identified in the resected specimens. Vancomycin and micafungin were initiated, in addition to meropenem, which had been administered preoperatively. These antibiotic agents were continued for 6 weeks and replaced with the following oral medications 34 days after surgery: minocycline, clindamycin, and fluconazole. The patient was discharged home. At 16 months postoperatively, she had no signs of recurrence.

## Discussion

sADF most commonly occurs between the third duodenal portion and proximal aortic anastomosis.^6^ Treatment requires enteric fistula repair and prosthesis replacement and has high mortality (40%).^3^ Early death is typically caused by persistent infection, infected aortic postoperative bleeding, and a worsening of the patient’s overall condition due to the high surgical invasiveness, generally resulting in multiple organ failure.^3^ Late death is most often caused by recurrent infection. Therefore, reliable infection control and less invasive surgery are required.

Prior work has reported that total prosthesis excision did not improve in-hospital mortality rates.^3^ Alternatively, partial excision limited to infected grafts with preservation of uninfected grafts can be considered for those with localized infection.^4^ However, the European Society for Vascular Surgery guidelines show that the recommendation class and evidence level for partial excision are IIb and C, respectively.^7^ Therefore, accurate identification of infected and uninfected grafts is critical.

FDG-PET/CT has shown potential utility for localizing and assessing the severity of prosthesis infection through FDG uptake.^5^^,^^8^ However, few studies have reported on the application of FDG-PET/CT in prosthesis preservation for sADF treatment. Goto et al^5^ reported successful aortoesophageal fistula treatment with prosthesis preservation using FDG-PET/CT, which revealed regions of focal high FDG uptake. In the present report, FDG-PET/CT was applied for diagnosis of the prosthesis infection and identification of the infection location and severity. From the FDG-PET/CT findings, we concluded that the infection sites were distributed around the sADF, occluded right medial limb, and ligated left lateral limb. Therefore, we resected the third portion of the duodenum and removed only the infected graft sites. This approach minimized the dissection and damage to surrounding tissue. The duodenum and infected partial graft were removed en bloc to avoid contamination of the intestinal fluid from the remnant graft.

Recurrent infection prevention is important in sADF treatment, for which the incidence of recurrence is influenced by the graft material. Rifampicin-soaked grafts have shown effectiveness against some gram-positive bacteria, although their effectiveness against methicillin-resistant *Staphylococcus aureus* and gram-negative bacteria is unclear.^9^ Bovine xenopericardial roll grafts have shown excellent outcomes against infection. One study reported that none of the patients treated with this graft developed recurrent infections or graft stenosis, calcification, or dilatation.^10^ However, complex shapes (eg, Y-shape) and length adjustment are difficult with bovine pericardium. We also considered other material, including allografts and autogenous deep vein. However, allografts are not easily accessible in Japan. Grafts of autogenous deep vein are effective in terms of long-term outcomes and duration of postoperative antibiotics.^11^ We needed to minimize the invasiveness of the procedure because of the patient's condition. Extra-anatomic bypass with aortic ligation is another strategy. However, it includes the risk of aortic stump rupture^12^ and the disadvantage of requiring long-term anticoagulation therapy. Therefore, we performed an aorto-uni type revascularization using a bovine pericardial roll and FF bypass. Another option for preventing recurrent infections is to use vascularized tissue flaps. The use of the omental flap has resulted in improved survival outcomes.^3^ However, for the present patient, an omental flap was not used because one had been created in her last surgery 7 years prior.

The anatomic limitations of the present case included the site of the sADF, which was located at the right limb and not at the proximal anastomosis, which would have required suprarenal dissection. However, it was a sufficient distance from the ampulla of Vater to staple and cut the duodenum. We consider that a further review of cases is necessary to determine the outcomes of partial graft resection guided by the FDG/PET-CT findings.

## Conclusions

In the present report, we describe the successful case of sADF repair with prosthesis preservation to reduce invasiveness, guided by the FDG-PET/CT findings. We have demonstrated the feasibility of FDG-PET/CT for improving the surgical strategies in high-risk patients with sADFs.
